# Bone marrow stromal cell-derived exosome combinate with fibrin on tantalum coating titanium implant accelerates osseointegration

**DOI:** 10.3389/fbioe.2023.1198545

**Published:** 2023-07-11

**Authors:** Jian-Tong Cui, Xin-Yuan Wang, Xiao-Dan Mu, Meng Huang, Ya-Di Wang, Qiang Luo, Hui-Xia He

**Affiliations:** ^1^ Department of Stomatology, The First Medical Center, Chinese PLA General Hospital, Beijing, China; ^2^ Shannxi Provincial Crops Hospital of Chinese People’s Armed Police Forces, Xian, China; ^3^ Medical School of Chinese PLA, Beijing, China

**Keywords:** exosome, fibrin, tantalum coating, osseointegration, bone marrow stromal cell

## Abstract

This study aims to present a sustainably releasing system of exosomes-fibrin combinate loaded on tantalum-coating titanium implants. We hope to investigate potential effects of the system on osseointegration between tantalum coating titanium implants and its surrounding bone tissue. Exosomes derived from rabbit bone marrow stromal cells (rBMSCs) and fibrin were deposited onto the micro-nanostructure tantalum coating surface (Ta + exo + FI) and compared to control groups, including tantalum coating (Ta), tantalum coating loaded exosomes (Ta + exo) and tantalum coating loaded fibrin (Ta + FI). The optimal concentration of loading exosomes, exosomes uptake capacity by BMSCs, and the effect of controlled-release by fibrin were assessed by laser scanning confocal microscope (LCSM) and microplate reader. The optimal concentration of exosomes was 1 μg/μL. Adhesion, proliferation, and osteogenic differentiation ability of BMSCs on different materials were assessed *in vitro*. Finally, osseointegrative capacity of Ta, Ta + exo, Ta + FI, Ta + exo + FI implants in rabbit tibia were respectively evaluated with histology and bone-implant contact ratio (BIC%). It was demonstrated that exosome sustained-release system with fibrin loading on the tantalum coating was successfully established. Fibrin contribute to exosomes release extension from 2d to 6d. Furthermore, Ta + exo + FI significantly promoted adhesion, proliferation, and osteogenic differentiation of BMSCs. *In vivo*, the implants in Ta + exo + FI group displayed the highest osseointegrative capability than those in other groups. It is concluded that this exosome delivery system on the implants may be an effective way for tantalum coating titanium implants to promote osseointegration between implant and its surrounding bone tissue.

## 1 Introduction

Osseointegration is a complex process involving a variety of cells, cytokines and related signaling molecules, during which interactions between cells and the material surface including early cells recruitment, proliferation and osteogenic differentiation are crucial. Recently, modification of dental implant surface with bioactive molecule delivery system to improve biological activity and enhance osseointegration has gained much interest. Bioactive molecules such as bone morphogenetic protein-2 (BMP-2), transforming growth factor-beta (TGF-β), and peptides were explored extensively to enhance surface biological activity and encourage reactions of implant and cells (HELLER et al., 2018; CIRERA et al., 2019). Growth factors, transformation factors and bioactive peptides loaded on the implant surface could promote bone marrow stromal cells (BMSCs) adhesion, proliferation and osteogenic differentiation on implant surface. Those biactive molecules could enhance osseointegration in animals by inhibiting bacteria and inflammation, as well as promoting BMSCs differentiation into osteoblasts while inhibiting osteoclasts formation (CHEN et al., 2015; CHO et al., 2016). However, limitations of bioactive molecules such as low stability, short half-life, and easy inactivation, hinder their application on implant surfaces. Under such circumstances, exosomes are gaining popularity due to their stable properties and double-layered phospholipid membrane structure.

Exosomes are extracellular vesicles secreted by various cells, with diameters ranging from 30 to 150 nm (PEGTEL and GOULD, 2019). Exosomes are highly prevalent in bodily fluids and are capable of transporting transfer proteins, mRNAs, or microRNAs to various cells. This process can modify gene expression and protein translation levels in the recipient cells, ultimately regulating biological activity of the target cells (CAMUSSI et al., 2013; GURUNG et al., 2021). Comparing to growth factors and bioactive peptides, exosomes exhibit better biocompatibility, lower immunogenicity, and better stability. Furthermore, numerous proteins, DNA, mRNA, and microRNA have been discovered in exosomes (DAI et al., 2020; ISAAC et al., 2021). The components and functions of exosomes differ from the source of cells (LI et al., 2016; SONG et al., 2019; ZHU et al., 2019). Exosomes derived from osteoclasts may stimulate osteoclast genesis, while those secreted by BMSCs not only can enhance osteoblast differentiation and mineralization (NARAYANAN et al., 2016; ZHAO L et al., 2018), but they also promote bone regeneration in rat fracture models (ZHANG L. et al., 2020). Researchers found that exosomes derived from BMSCs loaded on the titanium surface accelerated adhesion, proliferation and osteogenic differentiation of BMSCs (WEI et al., 2019; LAN et al., 2020). However, exosomes deposited on the metal surface may be rapidly released into the environment; therefore, prolonging function time and increasing duration to facilitate bone formation requires further studies. Additionally, whether exosomes loaded on dental implants surface could enhance osseointegration *in vivo* has not been reported.

Fibrin is a kind of reticular protein, whose structural properties can be controlled by changing fibrinogen or thrombin concentrations in precursor solutions (SPICER and MIKOS, 2010; KIM et al., 2013). Due to its biodegradable and biocompatible properties, fibrin has been widely utilized as a hemostat and sealant in surgical procedures (RODRIGUEZ-MERCHAN, 2017; FAWZY et al., 2021). Additionally, generated following tissue injury, fibrin serves as a natural scaffold that initiates hemostasis and provides an initial matrix for essential cellular processes such as adhesion, migration, proliferation, and differentiation (NOORI et al., 2017). The multiple interaction sites for cells and proteins make fibrin a bioactive matrix for drug, cell and biomolecular delivery systems.^13^ Being combined with metal or ceramic scaffolds, fibrin can exert good biological effects in bone tissue engineering (KALIA et al., 2006; KOPF et al., 2015). In this research, based on the micro-nano porous structure tantalum-coated titanium discs and dental implants in our previous study (CUI et al., 2023), exosomes derived from BMSCs were loaded onto the surface of tantalum coating, and fibrin was further compounded on the exosome-loaded surface. The aim of this study is to prepare an exosome sustained release delivery system, examine its effects on BMSC biological properties *in vitro*, and assess its osseointegration *in vivo*.

## 2 Methods

### 2.1 Cell culture and characterization of BMSCs

Rabbit BMSCs were isolated from ten 2-week-old New Zealand rabbit femurs, and the animal model was approved by the Animal Care Committee of Chinese People’s Liberation Army General Hospital (Approved No: 2021-x17-13). The BMSCs were incubated in Dulbecco’s modified Eagle’s medium (DMEM) supplemented with 10% fetal bovine serum (FBS) (Gibco, United States), 100 mg/L streptomycin and 100 U/mL penicillin and were cultured at incubator (5% CO_2_, 37°C). Passage 2–5 cells were used for following experiments. For adipogenic differentiation, cells are cultured in DMEM with FBS (10%), IBXM (0.5 mmol/L), dexamethasone (0.1 μmol/L), indomethacin (100 μmol/L), insulin (10 μmol/L), penicillin (100 U/mL) and streptomycin (100 mg/L). For osteogenic differentiation, cells are cultured in DMEM with FBS (10%), Vitamin C (50 μg/mL), β-sodium glycero-phosphate (20 mmol/L), Dexamethasone (100 nmol/L), penicillin (100U/mL), and streptomycin (100 mg/L). The induction medium was changed every 3 days. At day 12, cells were fixed and stained with Alizarin Red S for osteocytes, and Oil Red O for adipocytes.

The phenotype characterization of BMSCs were stained with rat anti-rabbit polyclonal antibodies CD34, CD45, CD90, and CD105 (eBioscience Inc., United States) and analyzed by flow cytometry (BD FACSCanto™, United States) for phenotypic characterization.

### 2.2 Exosomes isolation and characterization

The medium was replaced with exosome-depleted serum (10%) DMEM when BMSCs (passages 3–5) reached 85%–90% confluence, and the medium was collected 48 h(h) later. After collection, the medium was centrifuged at 4°C with the following steps to remove dead cells and cell debris: 300 g for 10 min, 2000 g for 10 min, 10000 g for 30 min. Supernatant was transferred to an ultracentrifuge tube, centrifuged at 100000 *g* for 70 min, and the pellet in the tube was the extracted exosomes.

The exosome morphology was observed using transmission electron microscopy under 100-kV (TEM, HITACHI H-7000FA, Japan). Additionally, the particle size and distribution of exosomes were analyzed by Zetasizer Nano (Malvern, United Kingdom) according to the manufacturer’s instructions. Antibodies against CD63 (ProteinTech, United States), CD9 (Abcam, United Kingdom), and GRP94 (Abcam, United Kingdom) were used to identify protein-level expressions by Western blot.

## 3 Establishment of exosome sustained release system by fibrin on Ta surface

### 3.1 Observation of morphology and adhesive capacity of exosome on Ta surface

The tantalum coating discs with a diameter of 10 mm and micro-nano structure were used in this study as described in our previous study (CUI et al., 2023). 100 μL 1 μg/μL exosome solution was loaded onto Ta surface for 12 h, then exosomes were fixed with 2.5% glutaraldehyde at 4°C for 2–3 h. The samples were washed with PBS for 10 min, briefly dehydrated in a graded series of ethanol (70%, 80%, 90%, 95%, and 100%) for 15 min each and dried by freeze dryer. Samples were sputter-coated with a gold layer and examined under SEM (S4800, Hitachi, Japan).

Ta samples were put to 48-well plates, and 100 μL 0.1 μg/μL, 0.5 μg/μL, 1 μg/μL, 1.5 μg/μL exosomes were added to wells respectively (n = 4). Excessive solution was removed after 12 h, and 100 μL of 2% sodium lauryl sulfate solution was added per well and shaken for 12 h. The concentration of exosomes in the solution was detected by microplate reader at wavelength 562 nm (TECAN, Switzerland).

### 3.2 Adhesion and morphology of fibrin on Ta surface

Fibrinogen was dissolved with deionized water to 80 mg/mL, and thrombin was dissolved with 40 mM CaCl_2_ solution to 500 IU/mL. Fibrinogen and thrombin solution were mixed with a ratio of 1:1 to obtain fibrin. Ta samples were put on a spin coater with a speed of 3,000 rpm, then 200 μL of fibrin solution was loaded on the surface of Ta. The protocols were repeated three times to acquire Ta + FI. Fibrin morphology on Ta surface was observed under SEM as above mentioned.

### 3.3 The process of exosome up taken by BMSCs

Mix the PKH67 and Diluent C to get working solution. Add exosomes to the working solution and further incubate for 10 min. After add 10 mL phosphate buffered solution to the solution, centrifuge the mixture with ultracentrifugation method at 100000 g for 1 h. Then the exosome stained with PKH67 were obtained. Ta samples were transferred to 48-well plates, and100 μL 0.1 μg/μL, 0.5 μg/μL, 1 μg/μL, 1.5 μg/μL PKH67-stained exosomes were added to the samples, respectively (n = 4). Excessive solution was removed after 12 h, and BMSCs in the plates at 5 × 10^4^ cells/well were inoculated in incubator for 12 h. Then the cells were fixed with 4% paraformaldehyde for 20 min and subsequently stained with DAPI. Exosomes and BMSCs on the surface were observed using laser confocal microscope (LCSM, Zeiss, Germany).

### 3.4 Detection the sustain-released effects of exosome combinated with fibrin on Ta surface

Fibrin was loaded on Ta + exo group samples to achieve Ta + exo + FI. Observation of exosome and fibrin morphology on Ta surface was done as described in 3.1. 200 μL of PBS was added into individual wells, and concentration of exosomes was determined at 37 °C. The OD value at 562 nm was determined by a microplate reader (n = 4).

## 4 The adhesion and proliferation ability of BMSCs on the Ta samples

BMSCs at passage 3 were seeded on Ta, Ta + exo, Ta + FI, and Ta + exo + FI samples, which were prepared in 24-well plates in Dulbecco’s modified eagle medium (DMEM, Gibco) with 10% FBS (Gibco) and 1% penicillin/streptomycin at 37°C, and 5% CO2 environment. To investigate the extent of cell adhesion, cell growth, and osteogenic differentiation potential in different samples, BMSCs were initially seeded at densities of 5 × 10^4^, 2 × 10^4^, and 1 × 10^5^ cells/well, respectively. A fluorescence microscope (Eclipse E600) was used to evaluate the morphology of adherent BMSCs cultured after 6 h. Cells were fixed with 4% formaldehyde for 30 min, washed twice with PBS, and then stained with rhodamine-Phalloidine and DAPI. Three representative images of each disc were captured and then analyzed for coverage area by ImageJ analysis software.

Cell Counting Kit-8 (CCK-8) assay was applied indirectly to quantify number of BMSCs seeded on Ta, Ta + exo, Ta + FI, and Ta + exo + FI samples after incubation for 1, 3, and 5 days. After 24 h, cell cultures were washed twice with PBS. 50μL of CCK-8 reagent (Dojindo, Japan) and 500 μL of culture medium were added to each well and incubated for 2 h at 37°C. 110μL of the mixture was transferred to 96 well plates and the absorbance at a wavelength of 450 nm was measured to examine the proliferation of BMSCs on different samples.

To observe the morphology and growth of BMSCs on the different samples, SEM was employed to visualize the BMSCs seeded on Ta, Ta + exo, Ta + FI, and Ta + exo + FI samples after 3 days of incubation. For preparation, samples were washed twice with PBS and fixed with 2.5% glutaraldehyde (Sigma, United States) for 2 h. After rinsing these samples three times with PBS for 10 min each, they were briefly dehydrated in a graded series of ethanol (70%, 80%, 90%, 95%, and 100%) for 30 min each and allowed to dry by Freeze dryer. Samples were sputter-coated with a gold layer and examined in an SEM (S4800, Hitachi, Japan).

## 5 The osteogenic differentiation ability of BMSCs on the Ta samples

### 5.1 ALP activity and alizarin red staining

BMSCs at passage 3 were seeded on different surfaces of the samples in 24-well plates. The induction media was composed of 10 mM β-glycerol phosphate, 50 μg/mL ascorbic acid, and 10 nM dexamethasone. After cultured for 24 h, the induction media was added to and replenished every 3 days. Extracellular matrix (ECM) mineralization and the alkaline phosphatase (ALP) activity of the cells were evaluated by ALP staining and ALP detection kit, respectively, after 14 days of incubation. Mineralization nodules were examined by alizarin red S after osteogenic induction for 21 days. In addition, all the samples were fixed in 4% paraformaldehyde for 30 min and stained with 2% alizarin red S for 6 h. These stained samples were desorbed with 10% cetylpyridinium chloride for 30 min and the absorbance was measured at a wavelength of 562 nm.

### 5.2 Real-time quantitative polymerase chain reaction (RT-qPCR)

The effects of Ta, Ta + exo, Ta + FI, and Ta + exo + FI on the osteogenic gene expression of COL-I, BMP-2, and Runx2 were assessed using Real-time quantitative PCR (RT-qPCR) with GAPDH as a reference marker. RNA of the BMSCs was collected at day 7 using the trizol reagent (Invitrogen, United States). The RNA was reversely transcribed to synthesized cDNA by the cDNA synthesis kit (Bio-Rad, United States). The equivalent cDNA was transcribed into mRNA using a CFX96 real-time thermocycler (Bio-Rad, United States). The mRNA expression level of COL-I, BMP-2, and Runx2 was analyzed using the ΔΔCt method. The primers sequences used in this study were displayed in Table 1.

**TABLE 1:** Primer sequences used for qPCR.

Gene	Primer sequence
Runx2	F:GCCACCACCCACTACCATAC
	R: GCT​TCC​ATC​AGC​GTC​AAC​AC
COL1	F: AGA​AAT​CCG​CTG​GAG​TCT​CG
	R: TCC​GTT​TTC​ACC​AGG​GCT​AC
GAPDH	F: GAA​GGT​CGG​AGT​GAA​CGG​AT
	R: TCT​CGC​TCC​TGG​AAG​ATG​GT
BMP-2	F: CCA​AGC​GTG​GAA​ACA​CAC​AGC​C
	R: GGC​TTT​GGA​ACT​CGC​CTG​ACT​G

## 6 Surgical implantation and histomorphometry

Twenty-four New Zealand rabbits (2.5–3 kg) were selected and randomly divided into four groups. The rabbits were put under general anesthesia by intramuscular injection of a mixture of xylazine hydrochloride injection and Midazolam (0.8 mL/kg). Four implants (Ta, Ta + exo, Ta + FI and Ta + exo + FI implants) were placed vertically in each rabbit’s tibia about 1 cm under the knee joint. Each animal was given intramuscular injection of 800,000 U penicillin per day for 3 days after surgery. Twelve rabbits in each group were euthanized after one and 3 months with lethal doses of sodium pentobarbital injected into a vein. Tibia tissue were separated into 2.5 cm×l.5 cm × 1.5 cm blocks and immersed in 4% polyoxymethylene.

The implant specimens were placed into a grinding plate with thickness of about 20 μm using a microtome (Leica, Germany) along its long axis. The samples were stained with Methylene blue acid fuchsin staining solution. Stained sections were then visualized under a light microscope (Eclipse E600). ImageJ was used to quantify the bone-to-implant contact (BIC%), which was then calculated as percentage of the length of the implant profile in direct contact with the bone surface to the length of the implant profile in bone.

## 7 Statistical analysis

All data in the experimental were expressed as mean ± SD. Statistical analysis was performed with unpaired two-tailed Student’s t-test for single comparisons with SPSS 18.0. Two-way ANOVA was used to compare data from more than two groups. Post hoc-tests were conducted. A value of <0.05 represents a statistically significant difference.

## 8 Results

### 8.1 BMSCs isolation and identification

**FIGURE 1:**
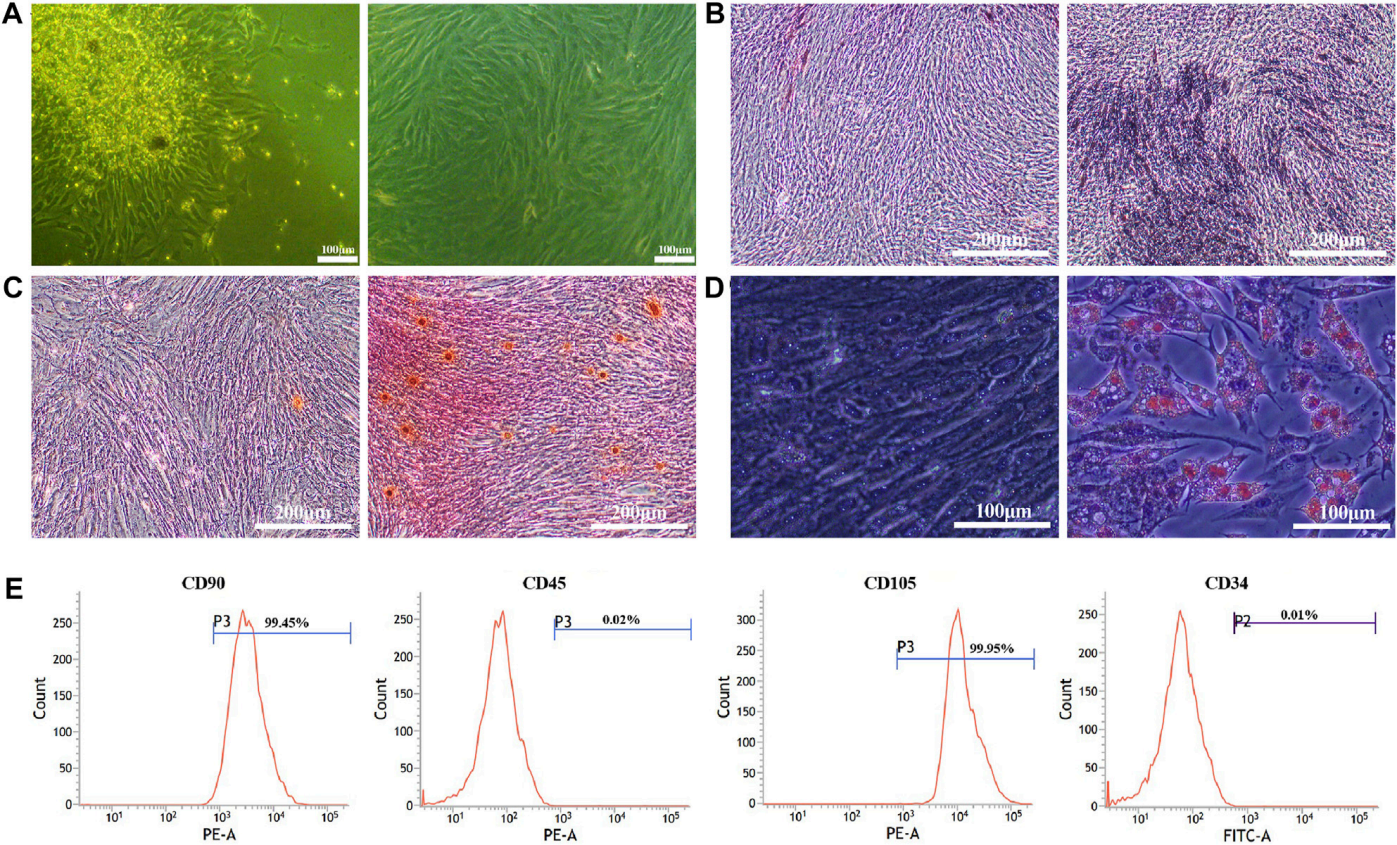
Isolation and characterization of BMSCs. **(A)**. BMSCs morphology at P0 (left) and P2 (right); **(B)**. ALP staining at 14 days (control group (left) and osteogenic induction group (right)); **(C)**. Alizarin red staining at 21 days (control group (left) and osteogenic induction group (right)); **(D)**. Oil red-O staining at 12 days (control group (left) and lipogenic induction group (right)); **(E)**. BMSCs surface markers of CD90 (99.45%), CD105 (99.95%), CD45 (0.02%) and CD34 (0.01%) detected by flow cytometry.

BMSCs were found to be extensive and characterized as short spindle, polygonal and colony growth in appearance (Figure 1A). After 14 days of osteogenic induction, the cells stained with ALP showed a massive blue-black staining in the induction group, while only a small amount of blue-black staining appeared in the control group (Figure 1B). Alizarin red staining of the cells exhibited a large number of red-stained calcified nodules in the induction group after 21 days of osteogenic induction (Figure 1C). After a 12-day lipogenic induction, oil red-O staining of the cells depicted that intracellular red bead-like lipid droplets were visible in the induction group, while no significant red fat droplets were found in the control group (Figure 1D). The results of flow cytometry showed (Figure 1E) that the surface markers of CD90 (99.45%) and CD105 (99.95%) on BMSCs were positive, and those of CD45 (0.02%) and CD34 (0.01%) were negative (Figure 1E).

### 8.2 Preparation and identification of exosome from BMSCs

**FIGURE 2:**
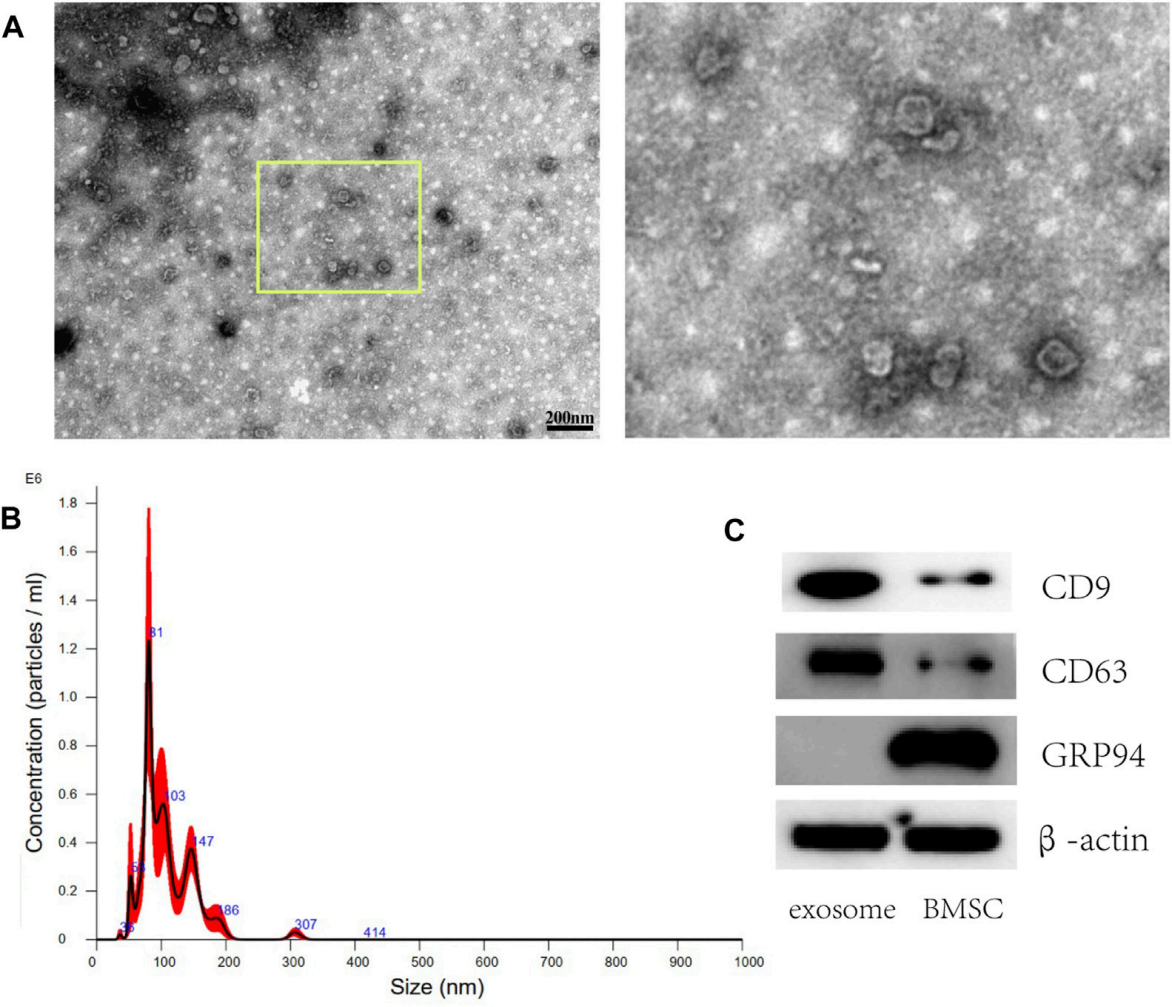
Characterization of exosomes derived from BMSCs. **(A)** Representative TEM images of exosomes isolated from BMSCs. Scale bar: 200 nm **(B)** The size distribution of exosomes was assessed using nanoparticle tracking analysis (NTA). **(C)** Western blot analysis of the exosome surface markers (CD9 and CD63 were positively expressed while GRP94 was negatively expressed).

Morphology of the exosomes isolated from BMSCs was observed by TEM as shown in Figure 2A, presenting as round shapes with double-layered membrane structure. The diameter ranges from 50 to 150 nm under the nanosight instrument (Figure 2B). Representative Western blot images revealed positively expressed surface markers of CD9 and CD63 on exosome from BMSCs while GRP94 related to endoplasmic reticulum was negatively expressed (Figure 2C).

### 8.3 The loading of exosome and fibrin on the porous Ta coating surface

**FIGURE 3:**
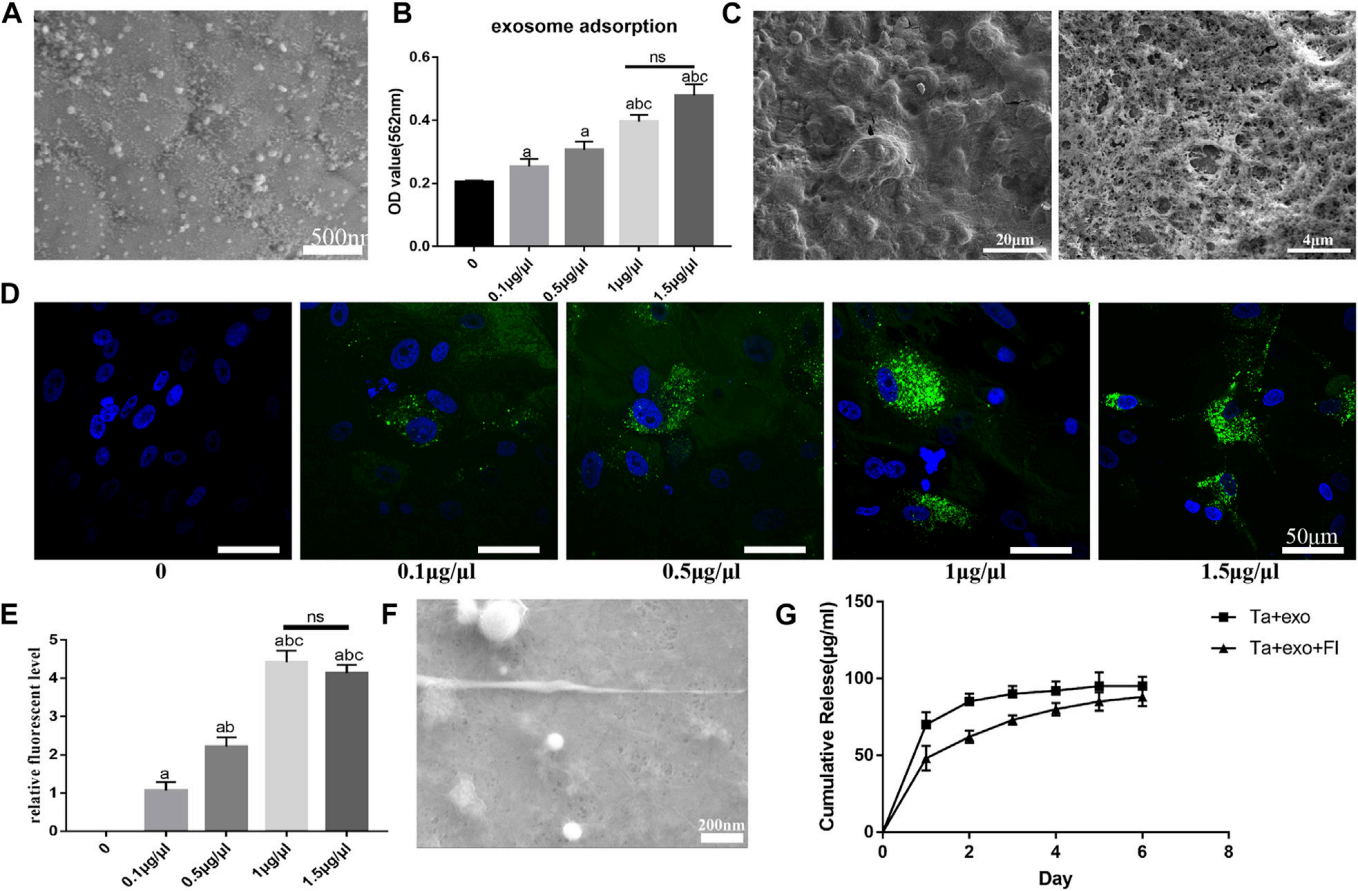
Establishment exosome sustain-released system with fibrin on Ta surface. **(A)** the morphology of exosomes on Ta surface observed by SEM; **(B)** the exosomes adsorption on Ta surface with different exosome concentration; **(C)** the morphology of fibrin on Ta surface observed by SEM; **(D)** the uptake of exosome (green) by BMSCs(blue) observed by LCSM; **(E)** the semi-quantitative analysis of exosomes in BMSCs; **(F)** the morphology of fibrin and exosome on Ta surface observed by SEM; **(G)** the exosome release on Ta surface with fibrin. (a. *p* < 0.05, compare with 0; b. *p* < 0.05*,* compare with 0.1 μg/μL; c. *p* < 0.05*,* compare with 0.5 μg/μL; ns. no significances).

According to SEM results, the small exosomes particles were fully adsorbed onto the surface of the various Ta coating after being infiltrated for 24 h (Figure 3A). The exosome concentration which was suitable for loading on the surface of Ta coating was further detected. The results (Figure 3B) demonstrated that the amount of exosome adsorbed on the porous Ta surface increased with the increase of exosome concentration when the concentration of exosome solution was lower than 1 μg/μL (*p* < 0.05). It showed no significant difference when the concentration rose higher than 1 μg/μL (1 μg/μL and 1.5 μg/μL) (*p* > 0.05). After loading fibrin on Ta surface, the fibrin reticulated and evenly covered Ta surface, with dendritic and reticular porous structure of varying diameters under SEM observation (Figure 3C).

To investigate uptake by BMSCs, the exosomes were labeled with the exosome marker PKH67 (green) and co-cultured with BMSCs (blue) for 12 h on the Ta samples. LCSM showed exosomes were concentrated around the nucleus, suggesting successful uptake of exosomes by BMSCs (Figure 3D). When exosomes were submerged in Ta samples at concentrations ranging from 0 to 1 μg/μL, quantity in cells increased as the concentration of loaded exosomes increased. The amount of exosomes did not rise considerably when the concentration of exosome solution was larger than 1 μg/μL, and there was no discernible difference between 1.5 μg/μL and 1 μg/μL (Figure 3E).

On Ta + exo + FI samples, granular and spherical exosomes coated with fibrin were found, and fibrin was equally covered on the surface of Ta and interconnected with Ta via an irregular network structure (Figure 3F). The results of exosome release on Ta + exo and Ta + exo + FI samples were shown in Figure 3G. On the first day, the exosome release in Ta + exo group was higher than that in Ta + exo + FI group. The release reached plateau stage on the second day in Ta + exo group, while that in Ta + exo + FI was slow and gradually stabilized at the sixth day. Finally, there was no significant difference in the amount of exosome release in both groups.

### 8.4 The adhesion and proliferation ability of BMSCs on the exosome-fibrin-Ta system

**FIGURE 4:**
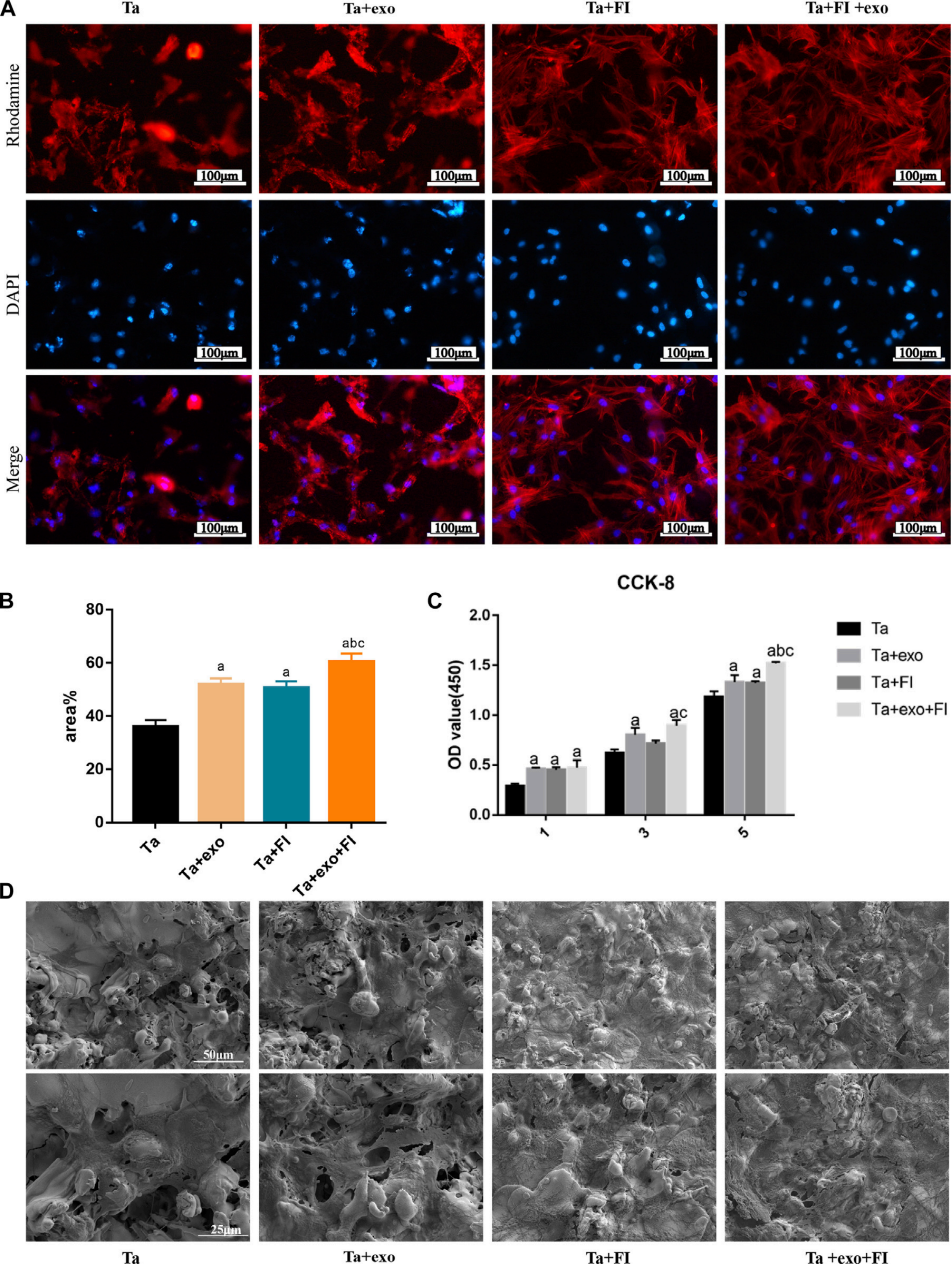
Adhesion and proliferation of BMSCs on different surfaces. **(A)**. Morphologies of BMSCs after 6 h of adhesion by fluorescence staining. **(B)**. Quantitative analysis of cell adhesion (n = 3); **(C)**. The proliferation of BMSCs on different samples after 1, 3, and 5 days (n = 3). **(D)**. SEM of BMSCs morphologies after 3d on different samples (a. *p* < 0.05, compare with Ta; b. *p* < 0.05*,* compare with Ta + exo; c. *p* < 0.05*,* compare with Ta + FI).

Fluorescence microscopy was utilized to observe the adhesion of cells on the surface of each specimen for 6 h (Figure 4A). Results indicated that number of adherent cells in the Ta + FI group and the Ta + exo group increased comparing to those of the Ta group in terms of sufficient stretching, increasing volume and long protrusions. Moreover, the cell number as well as protrusions that were intertwined into a network increased in Ta + FI + exo group and Image J analysis showed that the area occupied by cells per unit area increased significantly (*p* < 0.05) (Figure 4B).

The effects of exosome and fibrin on BMSCs proliferation on the Ta surface were detected by CCK-8 kit (Figure 4C) and the proliferation rate was determined by OD values obtained using a microplate reader. At day 1, 3 and 5, the OD values were higher in Ta + exo group than those in Ta group, suggesting significantly enhanced role of exosome on cell proliferation (*p* < 0.05). Interestingly, the OD values in Ta + exo + FI group were significantly increased than those of the other three groups, which suggested exosomes and fibrin could promote cell proliferation.

The morphology of BMSCs in each group at day 3 observed by SEM was shown in Figure 4D. Comparing to Ta group, more cells were observed in Ta + exo group, with a longer and irregular protrusion structure and enlarged cell body, which is closely connected with Ta surface. In the Ta + FI group, a massive of fibrin covering the pore structure of surface with irregular meshwork was observed, and the cell protrusions were anchored to the rough surface of tantalum metal and intertwined with the fibrin in a mesh shape. Comparing to the Ta + FI group, cell protrusions were further intertwined with the fibrin surface in a silk meshwork in Ta + FI + exo group, and matrix particles were secreted on the tighter structured membrane-like surface.

### 8.5 The osteogenic differentiation ability of BMSCs on the exosome-fibrin-Ta system

**FIGURE 5:**
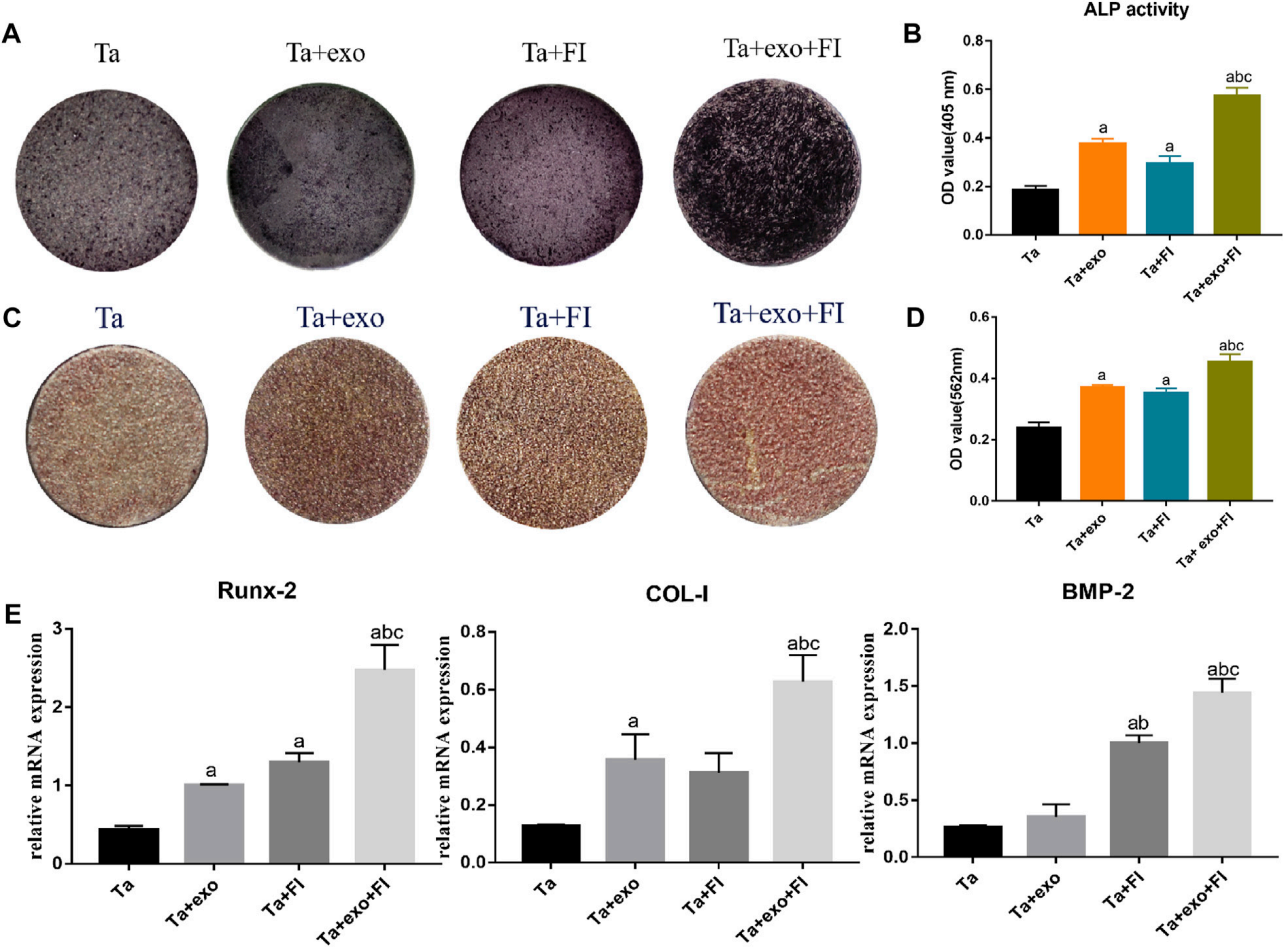
Osteogenic differentiation of BMSCs on different samples. **(A)**. ALP staining at 14d; **(B)**. ALP activities at 14 days (n = 3); **(C)**. Alizarin red S staining at 21d; **(D)**. Cell mineralization after culturing for 21 days (n = 3). **(E)**. The expressions of Runx2, COL-I, and BMP-2 gene of cells detected by RT-PCR at day 7 (n = 3). (a. *p* < 0.05, compare with Ta; b. *p* < 0.05, compare with Ta + exo; c. *p* < 0.05, compare with Ta + FI).

Blue-brown and black-brown stained particles of varying sizes were observed on the surface of the samples after osteogenic induction for 14 days and ALP staining (Figure 5A). The brown or brown-black staining area in the Ta + exo group and Ta + FI group was slightly larger than that in the Ta group, and the areas exhibited darker color and more cords, along with sheet-like connected particles in brown or black-brown color in Ta + exo + FI group. Results (Figure 5B) showed that the ALP activity level tested in the Ta + exo + FI group was significantly higher comparing to the other three groups (P < 0.05).

Alizarin red staining was performed to determine the effect of osteogenic induction on the surface of each coating in Figure 5C. Some areas on the surface of the Ta coating formed scattered brick red stained nodules. Compared with the Ta group, the coating surface of Ta + exo, Ta + FI group and Ta + exo + FI group was brick red, and the dyeing deepened sequentially and the red dyeing range increased. Semi-quantitative analysis revealed that the OD value of Ta + exo + FI group was higher than that of the other three groups (*p* < 0.05) (Figure 5D).

The mRNA expression of (Figure 5E) BMP-2, COL-I and Runx-2 in the Ta + exo group, Ta + FI group and Ta + exo + FI group was significantly higher than that in the Ta group (*p* < 0.05), among which the gene expression of Ta + exo + FI group was the highest, and the difference was statistically significant (*p* < 0.05), indicating that exosomes combined with fibrin could significantly promote the expression of osteogenesis-related genes.

### 8.6 The osseointegration ability of exosome-fibrin-Ta coating system modified implants *in vivo*

**FIGURE 6:**
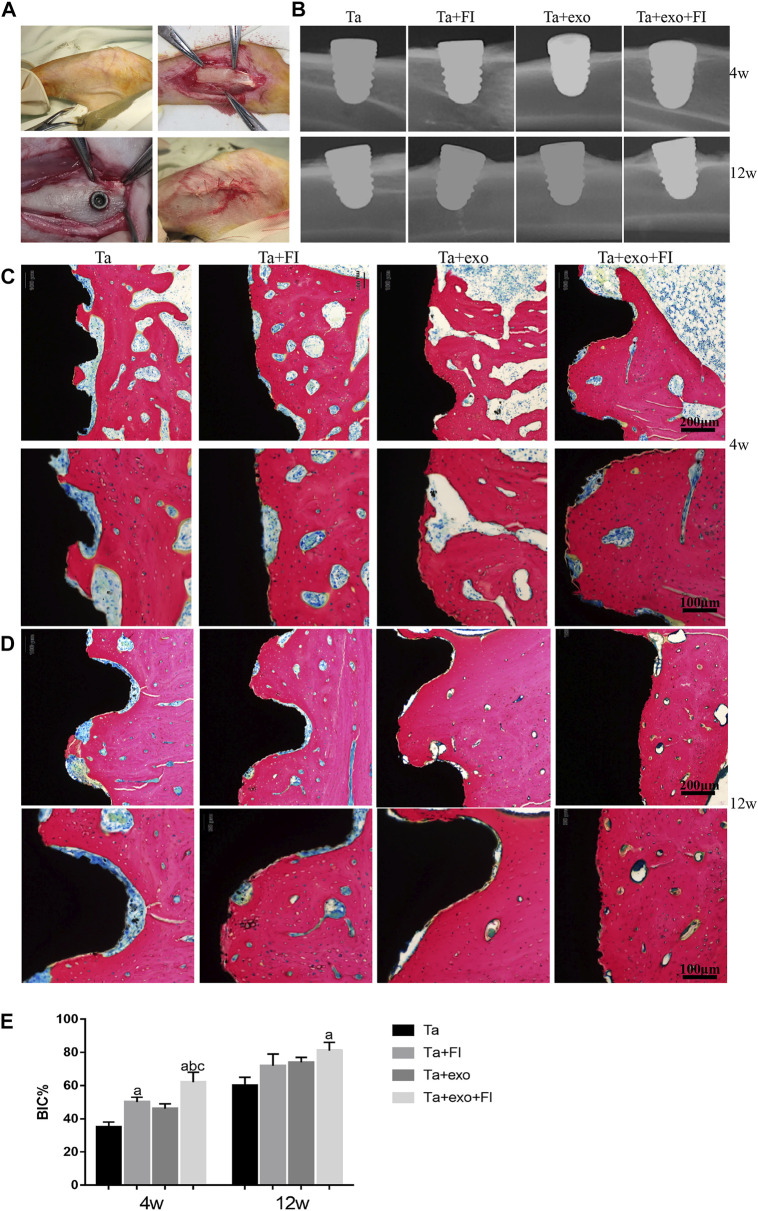
The ossteointegrative ability of different implants for rabbit tibia. **(A)**. The process of implantation; **(B)**. the X-ray after operation at 4 weeks and 12 weeks. **(C)**. Methylene blue-acid fuchsin staining at 4 weeks. **(D)**. Methylene blue-acid fuchsin staining at 12 weeks. (red: bone tissue; black: implant, white-blue gap between bone and implant: fibrous tissue). **(E)**. BIC% of the four groups after implantation (n = 3) **(C)**.(a. *p* < 0.05, compare with Ta; b. *p* < 0.05, compare with Ta+exo; c. *p* < 0.05, compare with Ta+FI).

Four types of implants (Ta, Ta + exo, Ta + FI, and Ta + exo + FI implants) were successfully placed into the rabbit tibia as shown in Figure 6A. The X-Ray results (Figure 6B) demonstrated that more bone tissue with high density was connected with implants at 12 weeks than that at 4 weeks. Methylene blue acid fuchsin staining at 4 weeks and 12 weeks was shown in Figure 6. An enlarged area of newly osteoid tissue (red) was observed around the implant (blank) in Ta + exo + FI group than those in Ta, Ta + exo and Ta + FI groups. More discontinuous gap between the bone and implant could be found in Ta, Ta + exo, and Ta + FI groups than the other two groups at 4 weeks. Furthermore, the Ta + exo + FI implant demonstrated more trabecular bones connecting directly with implants even at 12 weeks after implantation, and the white gap between bone and implant nearly disappeared. To quantitative compare osseointegration between different implants and tibia tissue, a calculational method of BIC% was used and the value of BIC% in the Ta + exo + FI group showed the largest bone connection ratio for implants (Figure 6E).

## 9 Discussion

The ideal implant surface should meet parameters such as mechanical stability, biocompatibility, and osteoinductivity, all of which have a significant impact on osseointegration (COELHO et al., 2015; PANAYOTOV et al., 2015; SHAH et al., 2019). One key technique to increase osseointegration is to load bioactive molecules onto implant surfaces to enhance osteoinductivity (PANAYOTOV et al., 2015). Exosomes have been shown to improve bone repair and regeneration in tissue engineering (WU et al., 2018), but few studies have concentrated on exosomes as bioactive molecules deposited on implant surfaces. Nonetheless, related research suggests that exosomes may be effectively loaded onto the surface of titanium discs. Wang et al. (PANAYOTOV et al., 2015) submerged titanium sheets in an exosome solution containing 4×10^10^ particles/mL overnight at 4°C and effectively loaded exosomes on the smooth titanium sheet surface. Lan et al. discovered that loading exosomes was simpler on acid-etched titanium sheets, which might be attributed to the rough surface structure and surface potential after acid etching (LAN et al., 2020). Previous research also found that exosomes exhibit a negative potential, since the surface potential impacted exosome loading (KANG et al., 2022). Our previous research revealed that the size of the fracture structure on the Ta coating surface ranged from 50 nm to 5μm, allowing for exosome accommodation. Furthermore, the lower absolute levels of negative potential on the Ta surface compared to the Ti surface were likewise favourable to exosome loading (CUI et al., 2023). In this study, we combined exosomes with Ta and assessed the exosomes’ adsorption capability on the Ta surface. By observing the uptake of exosomes by BMSCs and adsorption concentration, it was determined that 1 g/L was the optimal concentration for loading exosomes on the Ta surface. Furthermore, the lower absolute levels of negative potential on the Ta surface compared to the Ti surface were likewise favorable to exosome loading.

According to research, hydrogel may slow down the release of bioactive molecules loaded on the implant surface and extend its function period, hence increasing the amount and quality of new bone formation (WANG et al., 2017; MU et al., 2018). Hydrogel, for example, proved beneficial in delaying exosome release (LIU et al., 2018; HOLKAR et al., 2021; ZHANG et al., 2021). Zhang et al. injected exosomes derived from umbilical mesenchymal stem cells with hyaluronic acid hydrogel into a customized nano-hydroxyapatite/poly-ε-caprolactone (nHP) scaffold, and the sustained release of exosome was extended up to 20 days (ZHANG et al., 2021). Wei (WEI et al., 2019) et al. loaded exosomes onto the surface of polydopamine-coated titanium dioxide nanotubes, which progressively released exosomes within 12 h. Fibrin hydrogel could be injected as a liquid to fill arbitrary shaped holes and then solidify *in situ* to produce a dense network structure (NOORI et al., 2017). Furthermore, as a bioactive matrix, fibrin not only contains numerous interaction sites with cells and proteins, but it is also appropriate for cell and biomolecular delivery (AILISH BREEN and ABHAY, 2009). To avoid exosome destruction during the fibrin cross-linking process (JI et al., 2019), rather than suspending exosomes in fibrin precursor solutions, we initially loaded exosomes on the Ta surface before covering them with fibrin. This method not only lodged as many exosomes as possible into the pores of the Ta surface, but it also facilitated the penetration of fibrin into the pores to cover and wrap exosomes. Exosome release experiments revealed that fibrin increased the duration of exosome release from 2 to 6 days. Although some fibrin may break off during implant insertion owing to friction, the fibrin inside the pores was able to be preserved throughout implantation to limit the release of exosomes.

Material surfaces containing functional exosomes exhibit superior biocompatibility and osteoinductivity (WANG D. et al., 2022). Fan et al. (FAN et al., 2021)discovered that exosome-loaded polyetheretherketone (PEEK) stimulated BMSCs and RAW264.7 proliferation and stretch beyond unloaded PEEK. Cell proliferation and migration may be aided by BMSC-derived exosomes inducing fast activation of the extracellular regulatory protein kinase (ERK) and protein kinase B (AKT) pathways (ZHANG et al., 2018). Similarly, we discovered that the Ta + exo + FI surface boosted cell growth and adhesion area when compared to other groups. We report that exosomes are made to last longer by fibrin, and the combination of exosomes with fibrin increases cell proliferation even more. MSC exosomes included a slew of proteins and molecules (including osteogenesis-related proteins and microRNAs) that might play a role in the control of complicated signaling pathways and osteogenic differentiation (ZHAO L et al., 2018; ZHANG L. et al., 2020). Prolonging release of exosomes on the material surface seems to be more favorable to promoting the osteogenic differentiation *in vitro* and osseointegration *in vivo*. Kang (KANG et al., 2022) et al. manufactured a PLGA/Mg-GA2 scaffold and extended exosome release for up to 10 days, effectively stimulating new bone growth in rat cranial bone defect models. Similarly, the surface triggered osteogenic differentiation more efficiently in this study with the assistance of fibrin in extending exosomes release on the Ta + exo + FI surface. The extent of ALP staining, the number of formed calcified nodules, and the expression of BMP-2, COL-I and Runx-2 on Ta + exo + FI surface were significantly higher than those in other groups, suggesting that Ta treated with fibrin and exosomes effectively promoted the differentiation of BMSCs into osteoblasts. This might be due to the continual action of exosomes or fibrin’s favorable reactivity in promoting osteogenic differentiation. According to Kopf’s research, incubation cells on titanium sheets with dense fibrin resulted in higher expression of ALP and type I collagen, as well as better adhesion and early mineralization of human osteoblasts (KOPF et al., 2015). In another study, human osteoblasts showed more mineralization and expression of osteogenic genes on a fibrin-treated Ti surface than on Ti surface (RAO et al., 2019). Such finding was consistent with our findings of greater calcified nodule development in the Ta + FI group than in the Ta group, demonstrating that fibrin not only extended exosome release, but also synergistically functioned with exosomes in promoting osteogenic differentiation.

Exosomes derived from BMSCs demonstrated high biocompatibility and osteoinductivity *in vitro*, while also effectively promoted fracture healing and bone regeneration in tissue engineering in animals (WU et al., 2018; WANG D. et al., 2022; WANG W. et al., 2022). Currently, however, no study has focused on the *in vivo* osteointegrative impact of implant surface modification by exosomes. Due to the differences in animals and materials, no exact exosome concentration for implant surface was classified *in vivo* study. Therefore, according to the results *in vitro*, we fabricated Ta + exo + FI implants with 1 μg/μL exosome and implanted the implants to New Zealand rabbit tibia to analyze the effect of exosomes on osseointegration. The degree of integration between implant and bone is particularly important for clinical success. Better osseointegration results in greater stability and higher long-term success rate (HOWE et al., 2019; SHAH et al., 2019). Comparing to implants in Ta, Ta + FI and Ta + exo groups, those in the Ta + FI + exo group demonstrated tightest osseointegration with a large amount of bone matrix directly deposited on the implant surface. We further utilized BIC% to analyze osseointegration in different groups and found that the Ta + FI + exo group showed highest BIC% at 4w, indicating that exosomes and fibrin on Ta surface enhanced the osteogenic activity by inducing osteoblasts to secrete matrix and osteogenic mineralization. Such result was consistent with those obtained *in vitro*. It was revealed that fibrin and exosomes on tantalum coated implants coordinated *in vivo*. Delaying the release of exosomes enhanced bone formation and osseointegration in the early phases.

However, there are several limitations in this study. No comprehensive imaging analysis *in vivo* was done due to metal artifacts, further detection of linked new bone formation characteristics *in vivo*, such as immunofluorescence and bone density, is required. What’s more, the clinical application of exosome is an area that requires more investigations, and the use of exosome inclinical setting is currently limited due to a lack of universally accepted protocols for their isolation, separation, delivery, storage, and standardization of the optimal therapeutic dose (THERY et al., 2018; ZHANG Y. et al., 2020). For this study, it is essential to develop strategies to sustain exosome activity over an extended period to facilitate their clinical translation. At present, storing exosomes at −80 °C is recommended, although this approach is not generally employed due to the high expense of storage and shipping. As a result, academics have proposed alternate techniques. Charoenviriyakul et al. (CHAROENVIRIYAKUL et al., 2018) found that freeze-dried exosomes can be stored at room temperature while maintaining their key properties such as vesicle size, protein content, structural integrity, biological activity, and pharmacokinetics of transported drug molecules. From this point of view, compared to cryogenic freezing and gel preservation, freeze-drying is advantageous for the commercialization of exosome therapeutic drugs. Our next objective is to engineer, freeze-dry, and load exosomes onto implant’s surface to validate their effectiveness and facilitate their translation into clinical use. Furthermore, the role of exosome in promoting implant osseointegration on material surfaces has to be investigated further in future studies.

## 10 Conclusion

In this study, we successfully loaded exosomes and fibrin on the surface of tantalum coating. The loaded fibrin effectively slowed down release of exosomes, and the exosome-fibrin combination on the tantalum coated surface effectively improved BMSC adherence, proliferation, and osteogenic differentiation. The allosteric effect of exosome and fibrin on osseointegration was also confirmed in animal experiments. Loading exosome and fibrin on the surface of tantalum coating implants is an effective way to modify the implant surface and endow the implant surface with biological activity to improve osseointegration in the treatment of peri-implanttitis and other issues.

## Data Availability

The original contributions presented in the study are included in the article/Supplementary Material, further inquiries can be directed to the corresponding authors.
